# Nursing Home Staff’s Perceptions of Healthcare Decision Making for Unbefriended Residents

**DOI:** 10.1093/geroni/igaa057.1237

**Published:** 2020-12-16

**Authors:** Hyejin Kim, Molly Perkins, Thaddeus Pope, Patricia Comer, Mi-Kyung Song

**Affiliations:** 1 Chung-Ang University, Seoul, Republic of Korea; 2 Emory University, Atlanta, Georgia, United States; 3 Mitchell Hamline School of Law, Saint Paul, Minnesota, United States; 4 A.G. Rhodes Health & Rehab, Atlanta, United States

## Abstract

‘Unbefriended’ adults are those who lack decision-making capacity and have no surrogates or advance care plans. Little data exist on nursing homes (NHs)’ healthcare decision-making practices for unbefriended residents. This study aimed to describe NH staff’s perceptions of healthcare decision making on behalf of unbefriended residents. Sixty-six staff including administrators, physicians, nurses, and social workers from three NHs in one geographic area of Georgia, USA participated in a 31-item survey. Their responses were analyzed using descriptive statistics and conventional content analysis. Of 66 participants, eleven had been involved in healthcare decision-making for unbefriended residents. The most common decision was do-not-resuscitate orders. Decisions primarily were made by relying on the resident’s primary care physician and/or discussing within a facility interdisciplinary team. Key considerations in the decision-making process included “evidence that the resident would not have wanted further treatment” and the perception that “further treatment would not be in the resident’s best interest”. Compared with decision making for residents with surrogates, participants perceived decision making for unbefriended residents to be equally-more difficult. Key barriers to making decisions included uncertainty regarding what the resident would have wanted in the given situation and concerns regarding the ethically and legally right course of action. Facilitators (reported by 52 participants) included some information/knowledge about the resident, an understanding regarding decision-making-related law/policy, and facility-level support. The findings highlight the complexity and difficulty of healthcare decision making for unbefriended residents and suggest more discussions among all key stakeholders to develop practical strategies to support decision-making practices in NHs.

